# Dietary glucosinolates derived isothiocyanates: chemical properties, metabolism and their potential in prevention of Alzheimer’s disease

**DOI:** 10.3389/fphar.2023.1214881

**Published:** 2023-07-17

**Authors:** Farhana Khan, Abhishek Joshi, Hari Prasad Devkota, Vetriselvan Subramaniyan, Vinoth Kumarasamy, Jaya Arora

**Affiliations:** ^1^ Laboratory of Bio-Molecular Technology, Department of Botany, Mohanlal Sukhadia University, Udaipur, Rajasthan, India; ^2^ Graduate School of Pharmaceutical Sciences, Kumamoto University, Kumamoto, Japan; ^3^ Department of Pharmacology, Center for Transdisciplinary Research, Saveetha Dental College, Saveetha Institute of Medical and Technical Sciences, Saveetha University, Chennai, Tamil Nadu, India; ^4^ Department of Parasitology and Medical Entomology, Faculty of Medicine, Universiti Kebangsaan Malaysia, Kuala Lumpur, Malaysia

**Keywords:** Alzheimer’s disease, isothiocyanates, amyloid β, phosphorylated tau, glucosinolates

## Abstract

Alzheimer’s disease (AD) is the most prevalent form of dementia affecting millions of people worldwide. It is a progressive, irreversible, and incurable neurodegenerative disorder that disrupts the synaptic communication between millions of neurons, resulting in neuronal death and functional loss due to the abnormal accumulation of two naturally occurring proteins, amyloid β (Aβ) and tau. According to the 2018 World Alzheimer’s Report, there is no single case of an Alzheimer’s survivor; even 1 in 3 people die from Alzheimer’s disease, and it is a growing epidemic across the globe fruits and vegetables rich in glucosinolates (GLCs), the precursors of isothiocyanates (ITCs), have long been known for their pharmacological properties and recently attracted increased interest for the possible prevention and treatment of neurodegenerative diseases. Epidemiological evidence from systematic research findings and clinical trials suggests that nutritional and functional dietary isothiocyanates interfere with the molecular cascades of Alzheimer’s disease pathogenesis and prevent neurons from functional loss. The aim of this review is to explore the role of glucosinolates derived isothiocyanates in various molecular mechanisms involved in the progression of Alzheimer’s disease and their potential in the prevention and treatment of Alzheimer’s disease. It also covers the chemical diversity of isothiocyanates and their detailed mechanisms of action as reported by various *in vitro* and *in vivo* studies. Further clinical studies are necessary to evaluate their pharmacokinetic parameters and effectiveness in humans.

## 1 Introduction

In the past few decades, owing to healthy habits and general improvements in lifestyle and medication, life expectancy has substantially increased; however, the prominent upward shift in age distribution has increased the prevalence of chronic diseases, including Alzheimer’s disease (AD). AD slowly affects the brain and exhibits clear pathological changes in the hippocampus, the centre of memory and learning (Zhang et al., 2020). In AD, the propensity of neurotoxic proteins to form template or oligomers is higher and accelerates the conversion and aggregation of endogenous proteins, which eventually convert into fibrils (Schaffert and Carter, 2020). It can be sporadic or familial and AD cases are sporadic in most instances (Dorszewska et al., 2016). Disease modifying treatments primarily focused on reducing amyloid beta (senile plaques, Aβ) and tau (neurofibrillary tangles) load in the brain (Cammisuli et al., 2022). Despite many costly clinical trials ranging from pharmacological to hormonal treatments and immunotherapy, not even a single drug produced clinically significant results due to suboptimal dosing of drugs, unavailability of reliable biomarkers for early diagnosis and more specifically lack of detailed mechanistic approaches (Lashley et al., 2018; Loewenstein, 2022). The existing medication exert only moderate reduction of symptoms; therefore, AD remains symptomatic and can be controlled and prevented but uncured (Fernández and Ribeiro, 2018).

According to the World Alzheimer Report (2018), there are 50 million people living with dementia worldwide, of which 70–80 percent are AD patients, and by 2050 these numbers will be more than triple to 152 million (Patterson, 2018). From the data provided by the World Health Organization (WHO), it is an epidemic worldwide and has become a global burden (Cao et al., 2020). Death from AD has increased 123 percent between 2000–2005 and more than 60 percent cases are from low to middle income countries (Patterson, 2018). At the beginning of 21st century, AD remains a major biomedical challenge. Pharmaceutical companies and neurobiologists around the world are doing their efforts to develop novel FDA approved drugs such as acetyl cholinesterase (AChE) inhibitors (Donepezil, Rivastigmine and Galantamine) and NMDA (n-methyl D aspartate) receptor antagonist (Memantine) but they showed several side effects in phase II and III clinical trials. Common adverse effects of AChE inhibitors are diarrhea, nausea, vomiting, bradycardia, muscle twitching nightmares, etc., and memantine includes dizziness, headache, and lethargy (Ettcheto et al., 2018; Schneider, 2022).

The discovery of new natural pharmacologically active compounds is a widely growing field, as the synthesis of most the biomolecules is tough task (Ramawat and Arora, 2021). Consumption of antioxidant rich food and vegetables might improve brain function, minimize the possibilities of cognitive impairment, retard the process of aging, subsequent oxidation, and disease progression (Andrade et al., 2019). It is clinically proven that they enhance cellular metabolism and nourish brain cells; this safeguarding impact is more potent when isothiocyanates (ITCs) rich fruits and vegetables are specifically consumed (Esteve, 2020; Kamal et al., 2022). The propitious attributes of fruits and vegetables are related to their nutritional and functional components like minerals, vitamins, antioxidants and polyphenols. All of these molecules are found in cruciferous vegetables, however, the sulfurous compound GLCs that give them their distinctive pungent aroma and flavour set them apart. GLCs are stable chemically but biologically inactive and remain sequestered within plant compartment (Verkerk et al., 2009; Alexandre et al., 2020). Tissue damage and chewing are the main causes that lead to the formation of biologically active derivatives of GLCs such as ITCs by enzyme hydrolysis, which directly and indirectly regulate their activity and have been demonstrated to exert neuroprotective properties through multiple mechanisms (Tian et al., 2018).

Generally, there are three major hypothesis, i.e., AChE, amyloid, and tau, which are primarily implicated in Alzheimer’s disease management and prevention. Beside them, neuroinflammation is another important response target involving biochemical events activating resident cells of the central nervous system (CNS), which may induce the entire process of AD. It is initiated by aberrant astrocytes and microglial activation, which leads to the release of different inflammatory mediators such as nitric oxide (NO), prostaglandin E2 (PGE-2), reactive oxygen species (ROS), cytokines and chemokines (Kraft and Harry, 2011). Furthermore, it elevates the level of proinflammatory cytokines such as tumor necrosis factor (TNF-α), interleukin-1β (IL-1β) and interleukin-6 (IL-6), which are responsible for neuronal death (Xia et al., 2015). Controlling microglia and astrocytes activation can therefore be a therapeutic approach in the prevention and management of AD. Recently, it has been shown that ITCs possess neuroprotective effects through the modulation of different signalling pathways (Latronico et al., 2021). In oxidative stress and inflammation control, nuclear factor-kβ (NF-kβ) and nuclear erythroid related factor 2 (Nrf2) are two main regulators (Fão et al., 2019). They may primarily be attributed to their peculiar ability to activate the Nrf2/ARE pathway (Giacoppo et al., 2015). ITCs significantly decrease NF-kβ translocation with the inhibition of proinflammatory cytokines (Latronico et al., 2021). Hydrogen sulphide (H_2_S) is another important signal molecule in CNS; it could represent an intriguing strategy for the treatment of neurodegenerative diseases (Tabassum and Jeong, 2019; Sharif et al., 2023). Beside this, it also play a key role in many aspects of human health like in antiproliferation, cardioprotection, chemoprevention, etc. (Martelli et al., 2020). It also interacts with redox system regulating cellular oxidative stress and ROS (Kabil and Banerjee, 2010). There is a strong relationship between H_2_S and aging, as consistent significant decline of H_2_S levels has been observed in AD patients (Disbrow et al., 2021). H_2_S is a relevant player accounting for different biophysiological effects of Brassicaceae plants, for example, Allyl isothiocyanate (AITC) from black mustard (*B. nigra*), benzyl-ITC from garden cress (*Lepidium sativum*), erucin form *Eruca* sp.*, B. oleirecia*, etc. and 4-hydorxybenzyl-ITC from white mustard (*B. alba*) are some important naturally occurring ITCs. Among these selected ITCs, benzyl ITC is the most effective H_2_S donor, exhibiting remarkable H_2_S release followed by AITC (Citi et al., 2014). Recently, available literature clearly demonstrated that the role of natural ITCs as H_2_S donor (Martelli et al., 2020). It is a pleiotropic mediator that affects different element in inflammatory cascade specially NF-kβ and Nrf2 signalling (Zhao et al., 2023).

Another important effect of ITCs is apoptotic suppression as they can intervene and arrest the mitochondrial apoptotic pathway (Dinkova-Kostova and Kostov, 2012). Deposition of Aβ and hyperphosphorylated tau proteins is a crucial event in AD as pathology several studies demonstrated the pharmacological potencies of ITCs against these two hallmarks and their toxicity by intervene in its cascade such as APP cleavage, BACE1 expression, oligomerization of seeded proteins, phosphorylation and dephosphorylation assembly, etc. (Morroni et al., 2018; Asif et al., 2022). ITCs could therefore be considered as a promising source of medicine and for the treatment and management of AD. This review focuses on the knowledge regarding the direct and indirect mechanisms by which GLCs-derived ITCs intervene in inhibition of AChE, neurotoxic proteins (Aβ and tau) and neuroinflammation cascade.

## 2 Glucosinolates (GLCs) and isothiocyanates (ITCs)

### 2.1 Sources from foods

Glucosinolates (GLCs), a group of sulphur containing glycosides and their hydrolysis products, i.e., isothiocyanates (ITCs) are abundantly found in the family Brassicaceae which encompasses our daily vegetables including cabbage, broccoli, mustard, white radish, radish, kale, turnip, oilseed rape, collard greens, daikon, kohlrabi, wasabi, cauliflower, Brussels, etc. (Cancer et al., 2004; Shree et al., 2022). These metabolites distinguish them from other plant families and are responsible for pungent smell and bitter taste (Verkerk et al., 1998; Barba et al., 2016). Besides this, they are also found in *Moringa oleifera* (drumstick tree), a plant from the family Moringaceae; in contrast with other Brassicaceae plants, only aromatic GLCs have been identified in *M. oleifera* (Lopez-Rodriguez et al., 2020). More than 200 GLCs have already been characterized so far, although a small number of these compounds are present in closely related taxonomic groups and not all are present in plants that people consume (Fahey et al., 2001; Agerbirk and Olsen, 2012). Its content varies between different cultivars and plant species even in plant parts such as seeds, stems, roots, and leaves, while the highest amount is present in young tissues (Blažević and Mastelić, 2009). These variations arise from several factors (genetic, nutrient and environmental) and growth conditions (temperature, nutrient availability and water content).

### 2.2 Chemical properties

**FIGURE 1:**
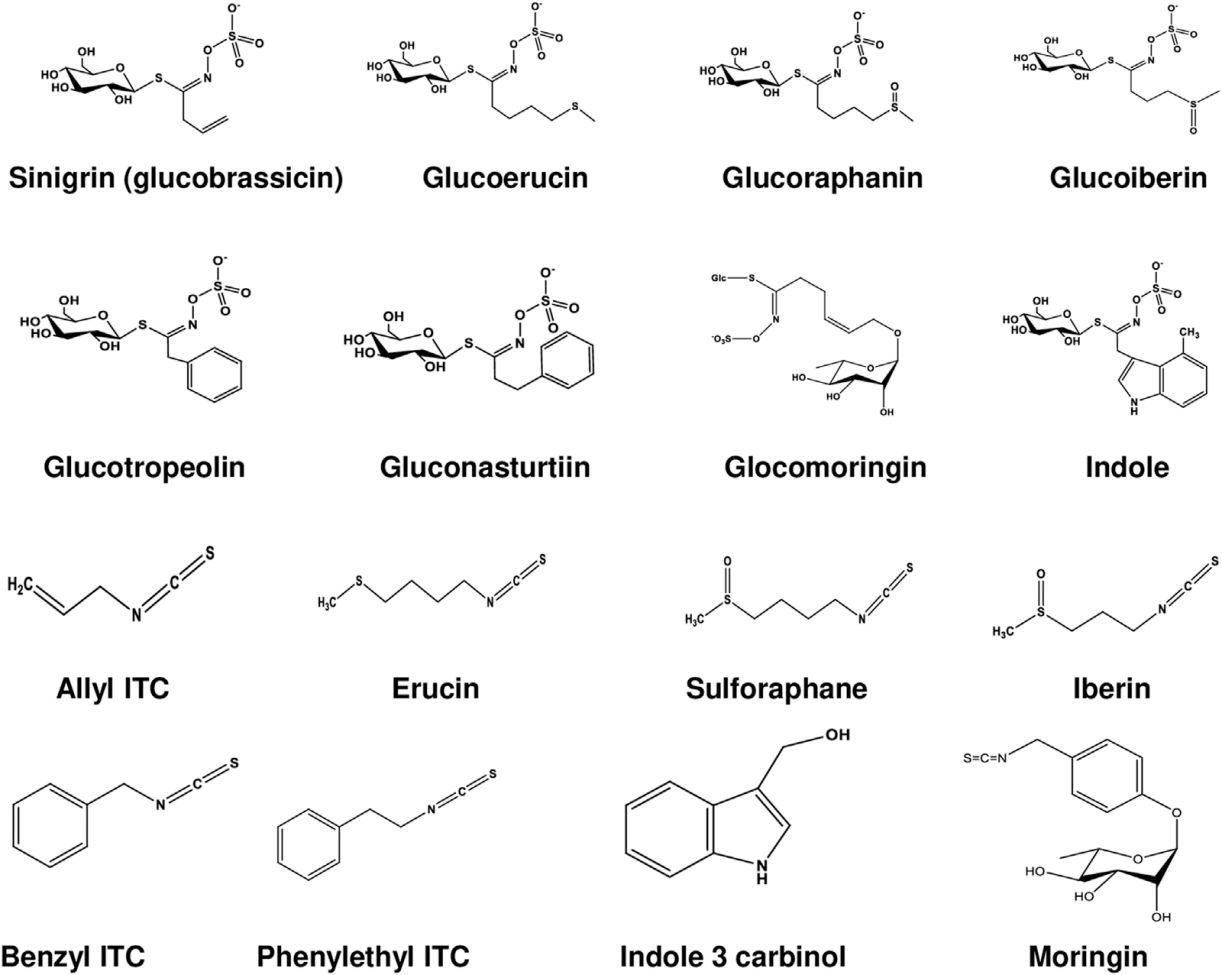
Chemical structures of glucosinolates and isothiocyanates.

GLCs are structurally thiohydroximates containing S-linked β-glucopyranosyl and O-linked sulfate residues with different side chains derived from amino acids (Agerbirk and Olsen, 2012). They are synthesized by different amino acid precursors such as phenylalanine, tryptophan, and methionine, which give rise to molecules with side chain R (Table 1; Figure 1). All known GLCs display structural homogeneity with different R groups in producing their corresponding ITCs responsible for various biological activities (Agerbirk and Olsen, 2012). On the basis of their side chain they are characteristically subdivided into three groups (Ali et al., 2018; Huke et al., 2021) as shown in Table 1: i) long chain length aliphatic; ii) short to medium chain length aliphatic (only C3 and C3 or C4 with C5) and iii) simple aryl aliphatic such as benzyl, phenyl, hydroxybenzyl GLCs; highly substituted aryl aliphatic such as dihydroxy, dimethoxy and trimethoxy benzyl GLCs. C3-C5 aliphatic GLCs are commonly found in *Brassica* species (Bennett et al., 2004).

**TABLE 1:** Trivial name, side chain structure and dietary plant source of Glucosinolates and Isothiocyanates.

GLCs trivial name	ITCs trivial name	Side chain name and structure of R group	Main dietary source
***Aliphatic group***			
Sinigrin (Glucobrassicin)	Allyl ITC	CH_2_ = CH-CH_2_^-^2-Propenyl	Cabbage, horseradish, wasabi, mustard Cartea and Velasco. (2008)
Glucoerucin	Erucin	CH_3_-S-CH_2_-CH_2_-CH_2_-CH_2_-4-Methyl thiobutyl	Turnip, kohlrabi, arugula, broccoli seeds Avato and Argentieri. (2015)
Glucoraphanin	Sulforaphane	CH_3_-SO-CH_2_-CH_2_-CH_2_-CH_2_-4-Methylsulphinylbutyl	Broccoli, cauliflower, kale, brussels sprout, cabbage Fahey et al. (2001)
Glucoraphenin	Sulforaphane	CH_3_-SO-CH = CH-CH_2_-CH_2_^-^4-Methylsulfinyl-3-butenyl	Radish, brussels sprout Fahey et al. (2001); Avato and Argentieri. (2015)
Glucoraphasatin	*Raphasatin*	CH_3_-S-CH = CH-CH_2_-CH_2_-4- methylsulfanyl 3-butenyl	Japanese Daikon Jaafaru et al. (2019b)
Glucoiberin	Iberin	CH_3_-SO-CH_2_-CH_2_-CH_2_^-^3-Methylsulfinylpropyl	Broccoli, cabbage Fahey et al. (2001); Cancer et al. (2004)
***Aromatic group***			
Glucotropaeolin	Benzyl ITC	C_6_H_5_-CH_2_^-^Benzyl	Wasabi and mustard Mithen et al. (2000); Verkerk et al. (2009)
Gluconasturtiin	Phenylethyl ITC	C_6_H_5_-(CH_2_)_2_^-^2-Phenylethyl	Watercress, radish, turnips, broccoli, kale Cartea and Velasco. (2008)
Glucomoringin	Moringin	C_13_H_15_O_5_^−^	Drumstick tree Lopez-Rodriguez et al. (2020)
**Indolyl ITC**			
Indol-3-yl-methylglucosinolate	Indole 3-carbinol	C_8_H_6_N-CH_2_OH 1H-Indol-3-yl-methanol	All vegetables Amarakoon et al. (2023)

**FIGURE 2:**
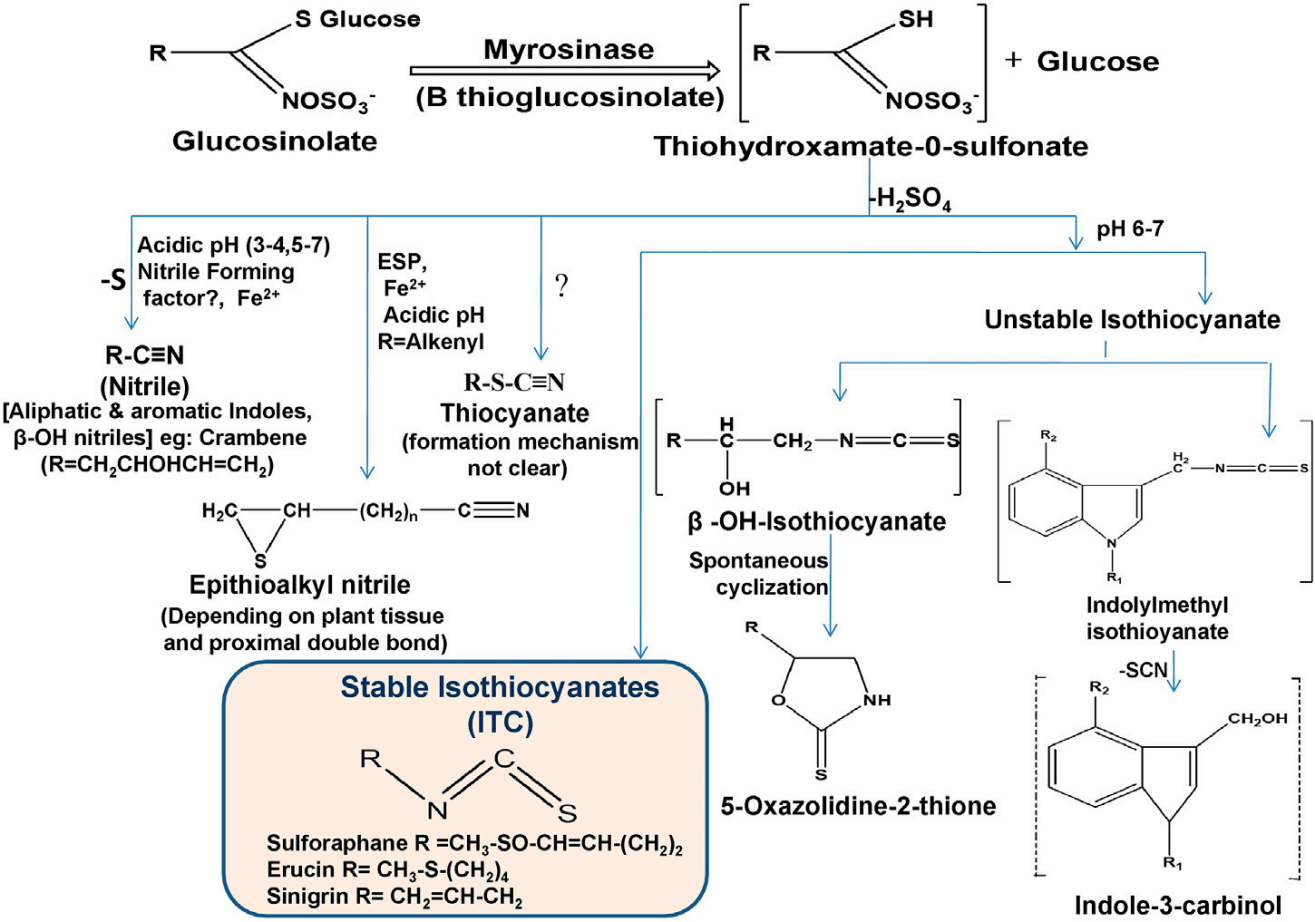
Enzymatic hydrolysis reaction of GLCs and their corresponding breakdown products (ESP; epithiospecifier protein).

ITCs are a specific type of compound derived from the hydrolysis of GLCs along with nitriles and thiocyanates. The entire conversion is catalyzed by endogenous myrosinase (thioglucoside glucohydrolase) enzyme released after chopping and chewing of raw vegetables or physical damage such as insect attack (Oliviero et al., 2018). Myrosinase reacts with GLCs by hydrolytically cleaving thio-linked glucose and forms active ITCs by an unstable intermediate thiohydroximate-O-sulfonate after immediate rearrangement depending on the corresponding substrate (GLCs), pH, temperature, epithiospecifier proteins (ESP), ferrous ions and thiocyanate forming proteins (TFP) (Sikorska-Zimny and Beneduce, 2021) as shown in Figure 2. Extraction and isolation of GLCs and their hydrolysis product ITCs are still challenging due to their sensitive nature. In recent years, different methods have been developed for the detection and quantification of GLCs and ITCs, mainly UHPLC-DAD-ESI-MS and HPLC-DAD-ESI-MS for GLCs (Devkota, 2020) and HPLC-DAD and UHPLC-HRMS/MS for ITCs (Karanikolopoulou et al., 2021). If myrosinase is denatured during ingestion, GLCs metabolism can also be triggered by gut microbiota (Luang-In et al., 2014). In such conditions, GLCs are absorbed in the stomach and then transit to the small intestine and colon where they hydrolyzed by microbiota (Barba et al., 2016). Long cooking time and high cooking temperature (>80°C) triggered myrosinase denaturation and significant GLCs and ITCs loss (more than 90%), but after ingestion, gut bacteria promote the conversion of GLCs into ITCs, which are then absorbed; therefore, a preferable method is steaming over boiling the raw food to minimize metabolite loss (Barba et al., 2016; Shakour et al., 2022).

### 2.3 Bioavailability of GLCs and ITCs

Bioavailability is an essential parameter that determines the action of metabolites. It represents absorption, distribution, metabolism, and excretion unlike drugs, where the oral concentration is predetermined. It depends on the number of food products, which is highly variable (Gupta and Robinson, 2017). It is evidently proved that ITCs are absorbed in higher amounts by passive diffusion from the gastrointestinal tract after ingestion into blood capillaries where they bind with free plasma proteins (thiocarbomylation) and pass into tissues cells where they affect their biophysiological mechanism (Kołodziejski et al., 2019). In a recent investigation, it was observed that broccoli converts gut microbiota to healthier profile, which coincides with myrosinase activity (Sikorska-Zimny and Beneduce, 2021). Most studies conducted among humans revealed that mercapturic acid pathway is involved in ITCs metabolism. One study using human urine explained that the ITCs can be absorbed indirectly through cylcocondensation determined by measuring plasma ITCs level after oral dose through high performance liquid chromatography with tandem mass spectrometry (HPLC-MS/MS) (Zhang and Zhang, 2017). Another study conducted on a rat model using radiolabel ITCs (14C) as an oral dose revealed the rapid absorption of ITCs, but the structure of individuals affects liposolubility (Chang et al., 2012). Both investigations observed that ITCs entered into enterocytes and glutathione S-transferase (GST) conjugated with glutathione favors internal accumulation and concentration gradient. Kidney and liver are involved in entire conversion because the liver contains high levels of glutathione and highest GST activity and plays a crucial role in xenobiotic detoxification by supporting accumulation of conjugated ITCs (Esteve, 2020). These conjugated ITCs are converted to mercapturic acid derivatives, which are implicated by the kidney due to the presence of γ-glutamyltranspeptidase (γ-GT), N-acetyltransferase (AT), and cysteinylglycinase (CGase), after they are excreted in urine (Shakour et al., 2022).

## 3 Role in neuroprotection, AChE inhibition, and neuroinflammatory mechanism

Neurons are the building blocks of the CNS, incapable of reproducing and replacing themselves. Several pathological disorders including AD are caused by the accumulation of reactive oxygen species (ROS) in cells (Deshmukh et al., 2017). The ability of a compound to possess anti-inflammatory, antioxidative, and/or antiapoptotic properties is currently used to establish neuroprotective and neuroinflammatory functions (Dinkova-Kostova and Kostov, 2012). ITCs were reported to play a protective effect in acute and chronic AD (Kamal et al., 2022). A variety of ITCs have been experimentally proven (Table 2) to reduce oxidative stress, inflammation, excitotoxicity, misfolded proteins, and mitochondrial dysfunction, and prevent programmed cell death (Connolly et al., 2021). Through the activation of ARE (antioxidant response element) driven genes, ITCs are strong Nrf-2 (nuclear factor erythroid factor 2) activators. They strongly suppress inflammation via NF-kβ (nuclear factor kappa light chain enhancer of activated β cells) pathway (Sita et al., 2016).

**TABLE 2:** The beneficial effects and mechanism of action of ITCs on various models of Alzheimer’s disease.

Compound or extract	Experimental model	Pharmacological effects	Mechanism of action	References
6-(Methylsulfinyl) hexyl ITC (6-MSITC)	*in vitro,* cell line	Slow down the progression of inflammation	Slow down pro inflammatory cytokines expression and increased Nrf2	Chen et al. (2010)
	*in vitro*, LPS activated murine macrophage RAW 264 cell line	Reduced neuroinflammation	Strongly suppressed COX-2, iNOS and cytokines and attenuated the expression of these factors	Uto et al. (2005)
	*in vivo,* murin AD model	Decreased apoptosis and neuroinflammation	Inhibited phosphorylation of ERK, GSK3, decreased inflammatory cytokines and activate of caspase	Morroni et al. (2018)
	*in vitro,* IMR-32 neuronal cell lines	Exerted neuroprotective effect by reducing oxidative stress	Targeted Nrf-2 mediated oxidative stress through changes in gene expression (DNA microarray)	Trio et al. (2016)
Phenethyl ITC(PEITC)	*in vitro,* cell line	Decreased inflammation	Initiated Nrf2, modulate Nrf2/AER signalling pathway	Qin et al. (2015)
	*in vivo,* transgenic mice model	Reduced inflammation, activated cytoprotective pathway	Restored Nrf2 expression	Boyanapalli et al. (2014), Dayalan Naidu et al. (2018)
	*in vitro* LPS-activated rat astrocytes	Anti-inflammatory	Downregulated MAPK/ERK signalling	Dayalan Naidu et al. (2018); Latronico et al. (2021)
Moringin	*in vivo,* rat model	Enhanced cognition	Modulated Nrf2/AER pathway and pro inflammatory biomarkers	Galuppo et al. (2015)
	*in vivo,* mouse model	Abolished inflammation	Modulated apoptotic pathway and downregulate pro inflammatory cytokines	Galuppo et al. (2014)
	*in vitro,* Aβinduced- SHSY5Y cells	Promoted neuronal repair and slowdown Alzheimer’s disease progression	Downregulated senescence, autophagy and mitophagy pathway	Silvestro et al. (2021)
	*in vivo,* lipopolysaccharide induced C57BL/6 mice model	Immunomodulatory and anti-inflammatory	Decreased pro inflammatory biomarkers (TNF-α, IL-1β, IL-6) in C2C12 myoblast, inhibited NF-kβ	Sailaja et al. (2022)
Erucin	*in vitro,* cell line	Stopped inflammation	Counteracted pro inflammatory markers expression, inhibited NF-kβ signalling pathway	Yehuda et al. (2012); Qin et al. (2015)
	*in vitro,* cell lines and *in vivo,* animal model	Decreased inflammation	Balanced Erk1/2, P38 and JNK signalling by Nrf2 pathway	Wagner et al. (2015)
	*in vitro,* LPS induced microglial cell line	Decreased inflammation	Decreased NO production, increased H_2_S levels	Sestito et al. (2019)
*Moringa oleifera* extract	*in vivo*, colchicine and ethyl Choline induced rat model	Reduced neuronal cell death, ameliorated memory impairment and improved spatial memory	Upregulated phase II antioxidant enzymes, SOD and catalase	Ganguly and Guha. (2008); Sutalangka et al. (2013)
	*in vivo*, cadmium and alcoholic beverage induced Wistar rats	Neuroprotection	Reduced the activated astrocytes in frontal cortex	Omotoso et al. (2019)
	*in vitro* primary hippocampal neurons culture	Promoted neurite outgrowth and promoted neuronal survival	Increased NSE, decreased GFAP	Hannan et al. (2014)
	*in vivo*, NDD/Al induced temporo-cortical degenerated mice model	Reduced neurodegeneration	AChE inhibitory activity	Ekong et al. (2017)
	*in vivo*, NDD/hippocampal neuro- degenerated rat model	Enhanced memory and cognition	Maintained neuron integrity and cholinergic transmission	Adebayo et al. (2021)
	*in vivo*, scopolamine induced mice model with spatial memory deficit	Improved spatial memory function	Altered the endogenous antioxidants, pro inflammatory mediators, elevatedAChE activity and promoted chromatolysis of cortical hippocample neurons	Onasanwo et al. (2021)
	*in vivo* lead acetate induced Wistar rat model	Ameliorated oxidative stress, inflammation and apoptosis	Protected neuronal cells via attenuation of NF-kβ signalling	Alqahtani and Albasher (2021)
	*in vivo,* CCl4 induced mice model	Modulated neuroinflammation and oxidative stress	Modulated TLR4/2MyD88/NF-kβ signalling	Mahmoud et al. (2022)
Sulforaphane	*in vitro,* human neuroblastoma cell line (SH-SY5Y)	Inhibited apoptosis	Modulated Bax/Bcl2	Lee et al. (2013)
	*in vitro,* murine neuroblastoma cell line (Neuro 2A and N1E-115)	Increased proteasome activity	Enhanced Nrf2 pathway	Park et al. (2009)
	*in vivo,* AlCl_3_ and D-galactose induced mice model	Ameliorated cognitive impairment	Modulated Nrf2/ARE pathway	Zhang et al. (2014)
	*in vivo* mice model	Reduced inflammatory markers in glial and hippocampal cells, protected neurons	ITH12674 (melatonin sulforaphan hybrid) induced Nrf2 and scavenged free radicals	Michalska et al. (2020)
	*in vivo,* scopolamine induced mice model (C57BL/6) and *in vitro* scopolamine treated primary cortical neurons	Improved memory, cognition and cholinergic neurotransmission	Inhibited Acetyl cholinesterase (AChE)	Lee et al. (2014)
	*in vitro,* Swedish mutant mouse model (N2a/APPswe cells)	Inhibited Aβ generated neuroinflammation and oxidation	Epigenetic modification of Nrf2	Zhao et al. (2018)
	*in vitro,* human THP-1 macrophages (induced by Aβ_1-42_)	Suppressed neuroinflammation	Downregulated NF-kβ pathway and preserved MERTK	Jhang et al. (2018)
	*in vitro,* amyloid induced microglial cells	Induced neuroinflammation	Increased microglial phagocytic activity	Chilakala et al. (2020)
	*in vitro,* dopaminergic SH-SY5Y human cells and LPS stimulated microglial BV2 cells	Prevented mitochondrial impairment and suppress neuroinflammation	InhibitedHO-1 enzyme	Brasil et al. (2023)
	*in vivo,* LPS induced rat model	Reduced inflammation	Suppressed LPS induced NF-kβ pathway, modulated TRAF6 and RIPI ubiquitination by cezanne	Wang et al. (2020)
Allyl isothiocyanate (AITC)	*in vitro,* neuroinflammatory model (NDD/LPS induced N2a neuroblastoma, BV2 murine microglia and C6 glioma cells)	Improved outgrowth of neurite and dysregulated apoptotic pathway	Suppressed NF-kβ/TNF-α/JNK signalling	Subedi et al. (2017)
	*in vitro,* cultured Schwann cells	Reduced neurogenic inflammation	Activated ROS dependent TRPA1	De Logu et al. (2022a)
	*in vitro,* murine RAW264.7 macrophages cell line, *in vivo* C57BL/6 mice	Suppressed inflammation	Decreased NF-kβ, downregulated pro inflammatory cytokine (IL-1β) and nitric oxide synthase, increased Nrf-2 and heme-oxygenase-1	Wagner et al. (2012)
	*in vivo,* cryogenic injury mice model	Increased plasticity markers level, regulate antioxidant genes	Decreased NF-kβ, GFAP, IL1β, IL-6, BBB permeability, increasing GAP43 and neural cell adhesion molecule	Caglayan et al. (2019)
Indole-3-carbinol (I3C)	*in vitro,* NDD/LPS induced BV-2 microglia (hyper activated)	Anti-apoptotic and anti-n -euroinflammatory activity, reduced microglial activation in hippocampus	Inhibited NF-kβ	Lee et al. (2014)
	*in vitro,* PC12 neuronal cells (NDD/glutamate excitotoxicity)	Inhibited apoptotic pathway	Inhibited caspase 8 and 3, scavenged ROS	Jeong et al. (2015)
	*in vivo,* mice model	Suppressed neuroinflammation and oxido-nitrosoactive stress in brain	Decreased BDNF, GSH, increased levels of nitrites, malondialdihyde IL-1β, TNF-α	Huang et al. (2022)

**FIGURE 3:**
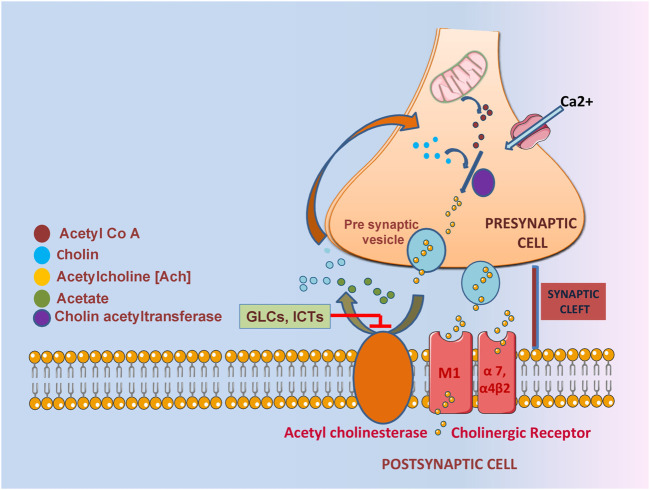
The role of GLCs derived ITCs in AChE inhibition characterized by impaired acetylcholine neurotransmission.

A deficient and non-equilibrium cholinergic neurotransmission is responsible for the pathophysiology of learning and memory resulting behavioral disturbance, progressive loss of cognition and daily routine function (Hoyer, 2004; Craig et al., 2011). In context with the cholinergic hypothesis, decreasing the amount of acetylcholine in the hippocampus and cerebral cortex leads to the dysregulation of ChAT and premature loss of basal forbidden cholinergic neurons (Burčul et al., 2018; Hampel et al., 2019). One of the most significant properties of ITCs is AChE inhibition implicated in acetylcholine neurotransmission (Figure 3). In one study, 11 different ITCs were evaluated for their AChE inhibitory and anti - inflammation properties; the most promising inhibitory activity among 11 ITCs was demonstrated by phenyl isothiocyanate and its derivatives. The most potent AChE inhibitory activity was shown by 2-methoxyphenyl ITC with IC_50_ value of 0.57 mM. Human COX-2 enzyme was also used to evaluate the anti-inflammatory activity, ranking phenyl ITC and 2-methoxy phenylITC as the most potent with 99% inhibition at 50 μM (Burčul et al., 2018). Moringine-specific benzyl ITC from *Moringa Oleifera* modulated the Nrf2/AER pathway, proinflammatory biomarkers, and apoptotic pathway in different mouse and rat models (Galuppo et al., 2014, (Galuppo et al., 2015). In another mouse model (LPS induced), it was found that ITCs effectively decreased TNF-α, IL-1β, IL-6 and inhibited NF-kβ (Sailaja et al., 2022). It also downregulated senescence as it promoted neuronal repair in *in vitro* Aβ induce SH5Y5Y cells (Silvestro et al., 2021).

Through different mechanisms (explained in Table 2), SFN prevented cognitive impairment, reduced the Aβ and tau biomarkers, oxidative stress, inflammation and neurodegeneration in experimental models (Kim, 2021). SFN was able to improve spatial and contextual memory through the Y-maze test and counteract the Aβ aggregate induced memory deficits induced by intracerebroventricular (ICV) injection in a mouse model (Kim, 2021). In the hippocampus and frontal cortex, SFN increased cholinacetyltransferase (ChAT) expression, decreased acetylcholine esterase (AChE) activity, and raised the level of acetylcholine (AChE) (Lee et al., 2014). In another study on a transgenic AD mouse model, it was observed that SFN not only reduced the production and deposition of Aβ plaques in the hippocampus and cerebral cortex but also it is associated with neurobehavioral deficit (Zhang et al., 2015; 2017). The neuroinflammatory inhibition is through the activation of Nrf2/HO-1 pathway and inhibition of JNK/AP-1/NF-Kβ by SFN. SFN significantly increased proteasome activity and enhance Nrf-2 pathway in murine neuroblastoma cell lines (Park et al., 2009). It also modulated the Nrf2/ARE pathway in an AlCl_3_-and D-galactose induced mice (Zhang et al., 2014).

Neurogenesis has been shown to be enhanced by AITC and PEITC. AChE inhibitory activity in AD revealed that PEITC inhibited the enzyme more effectively than benzyl ITC and AITC (Burčul et al., 2018). In another study, PEITC inhibited Akt activation, suppressed NO production through INF induction, and had an anti-inflammatory effect (Okubo et al., 2010). PEITC showed a protective effect by modulating the MAPK pathway (Ma et al., 2017). Experimental findings revealed that in LPS-induced inflammation model, AITC showed a neuroprotective effect mediated through downregulation of JNK/NF-kβ/TNF-α signaling (Subedi et al., 2017). It also activated ROS-dependent TRPA1 signaling, resulting in neurogenic inflammation reduction in cultured Schwann cells *in vitro* (De Logu et al., 2022a; De Logu et al., 2022b). PEITC decreased inflammation and activated the cytoprotective pathway in transgenic mice model by modulating Nrf2/AER pathway and restoring Nrf-2 expression (Boyanapalli et al., 2014; Dayalan Naidu et al., 2018). In another study using LPS-activated rat astrocyte culture, PEITC significantly downregulated MAPK/ERK signaling and influenced the inflammatory pathway (Latronico et al., 2021). Increasing evidences suggests that cytochrome p450 is fundamental for brain homeostasis and function while phase II enzyme such as glutathione *S*-transferase play a key role in redox homeostasis. Modulation of these enzymes can be achieved by ITCs, in the recent studies glucuronosyltranseferase expression increase by sulforaphane in HepG2 cells, in another study erucin and phenethyl ITC elevated glucuronosyltranseferase activity in rat liver slices (Abdull Razis and Mohd Noor, 2013).

*Moringa oleifera* extract (MOE) decreased the neuritis resulting from naturally occurring cellular injury, with the development of multipolar primary process (Hannan et al., 2014). It also suppressed oxidative stress, MDA, nitrite and TNF-α, increased SOD and inflammation and improved spatial memory and cholinergic neurotransmission by reducing AChE activity and loss of cortico-hippocampus neurons in rat model fed with *M. oleifera* seeds in dose dependent manner (Onasanwo et al., 2021). *Moringa oleifera* extract also scavenged free radicals produced by NO, iNOS and nitrotyrosine increase Nrf2 in LPS-activated macrophages and downregulated antioxidative genes; HO-1, GST-P1 and NQO- (Jaja-Chimedza et al., 2017). In another study, it significantly inhibited AChE and reduced neurodegeneration in an NDD/Al - induced temporocortical degenerated mice model (Ekong et al., 2017). *Moringa oleifera -* supplemented male Wistar rats showed improved memory when evaluated by the Morris water Maze test and significantly reduced AChE levels in brain tissues in a dose-dependent manner (Adebayo et al., 2021). In another observation, GMC-ITC treated neuronal cells (SH-SY5Y) significantly alleviate oxidative stress condition by reducing ROS level ((Jaafaru et al., 2019a). Glucomoringin ITC (GMC-ITC) isolated from *M. oleifera* seeds abrogated oxidative stress and showed neuroprotective activity against cytotoxic neuroblastoma cells (SH-SY5Y) induced by H_2_O_2_, gene expression study of detoxifying markers (phase II) by GMC-ITC revealed that all involved genes significantly express themselves. It also decreased the expression of NF-kβ and increased the expression of Ikβ, Nrf2, SOD-1, NQO1 and Nf-kβ respectively (Jaafaru et al., 2019b). *Eruca sativa* extract (ESE) with a high amount of erucin (ER) prevented cell death and degeneration induced by LPS in NSC-34 motor neurons exposed to LPS-stimulated macrophage cell culture medium by inhibiting FasL (tumor necrosis factor ligand superfamily number 6 expression) and suppressing pro-inflammatory mediators (attenuates TLR4, COX-2 expression of TNF-α level) (Gugliandolo et al., 2018). Erucin decreased inflammation in different cell line models, counteracted proinflammatory marker expression, and balanced Erk1/2, P38, and JNK signaling (Yehuda et al., 2012; Wagner et al., 2015). Indol 3 carbinol (I3C) is another promising candidate found in vegetables; it reduces the free radical production in neuronal cells (Mammana et al., 2019). It also showed the potent radical scavenging activity by chelating already produced free radical species (Giacoppo et al., 2015). In another study, it suppressed the expression of NO, COX-2, and iNOS in the brain, which prevented apoptosis and inflammation by inhibiting NF-kβ and IB phosphorylation (Kim et al., 2014). Furthermore, it decreased BDNF, GHS and increased TNF-α, IL1-β in mice model, it also helped in suppression of neurodegeneration (Huang et al., 2022). In another experiment, researchers explored the antioxidant and anti-inflammatory activity of SFN and ERN as H_2_S donor through the combination with rivastigmine in microglia and neuronal cell line (SH-SY5Y). Result revealed that both derivatives show significant antioxidant and anti inflammatory activities in microglial cell line, expression of antioxidant defense protein (GSH) was also induced in neuronal cell line. It significantly decreased the ROS production and NO release in microglial BV-2 cells. Further Erucin exerted a time dependent Nrf2 activation in SH-SY5Y cells (Sestito et al., 2019). When anti-inflammatory effect of erucin was evaluated in LPS-challenged umbilical vein endothelial cells (HUVECs), it significantly prevented the increase of ROS, TNF-α levels and decreased COX-2. It also induced NF-kβ (Ciccone et al., 2022).

## 4 Potential role of GLCs and ITCs against pathological hallmarks and their neurotoxicity

**FIGURE 4:**
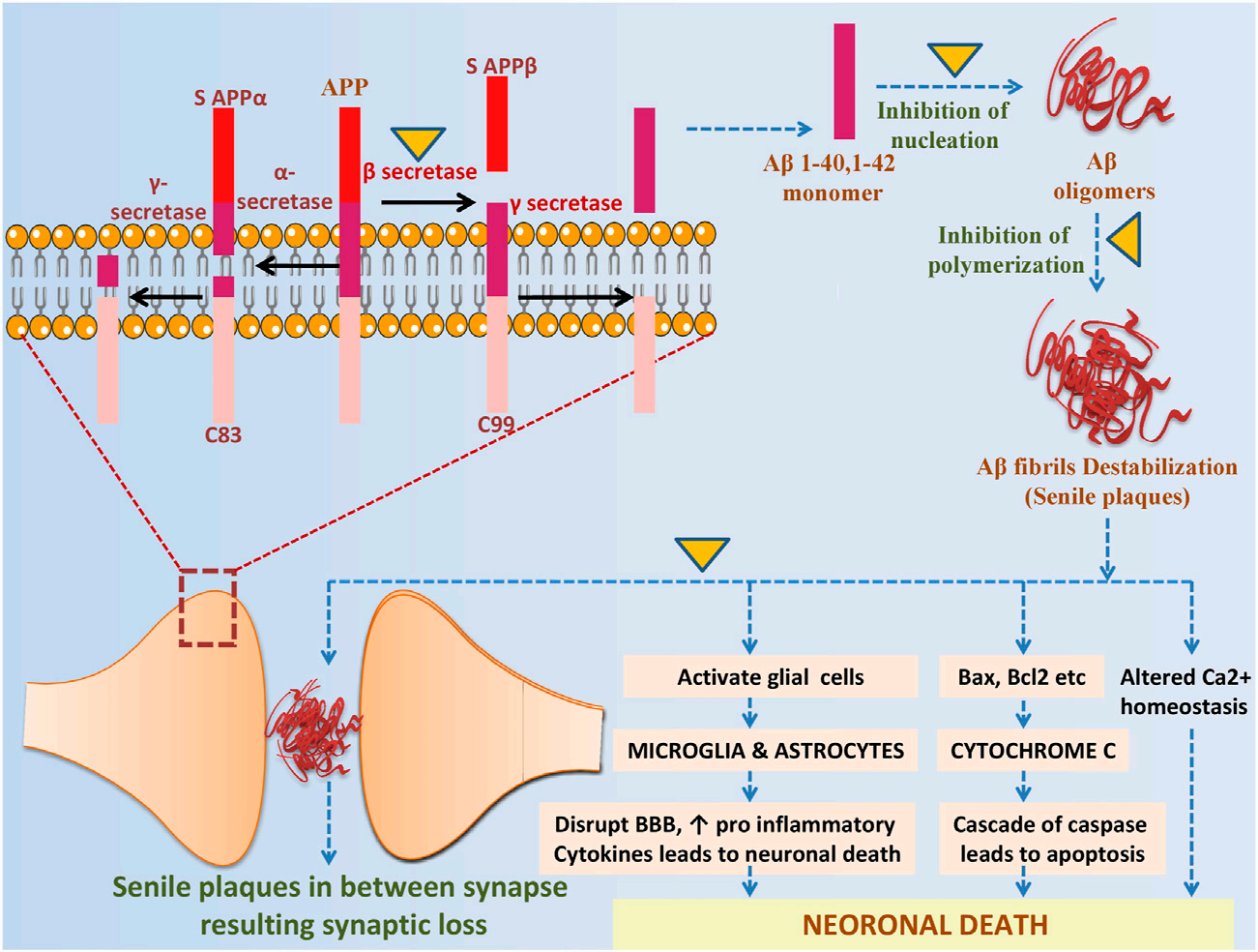
The potential role of ITCs in Aβ metabolism and related toxicity: sAPPα and C83 (membrane associated fragment) are formed by nonamyloidogenic pathway in which APP is cleaved by α-secretase, while in amyloidogenic pathway APP is cleaved by β-secretase producing S APPβ and C99 fragment, γ-secretase then processed the C99 and release Aβ. ITCs prevent from amyloidogenic cleavage by inhibiting β-secretase, further it inhibits nucleation, polymerization and plaques formation. It directly intervenes in Aβ induced neurotoxicity by altering Ca2+ homeostasis, downregulating cascade of caspase and in reducing inflammation.

**FIGURE 5:**
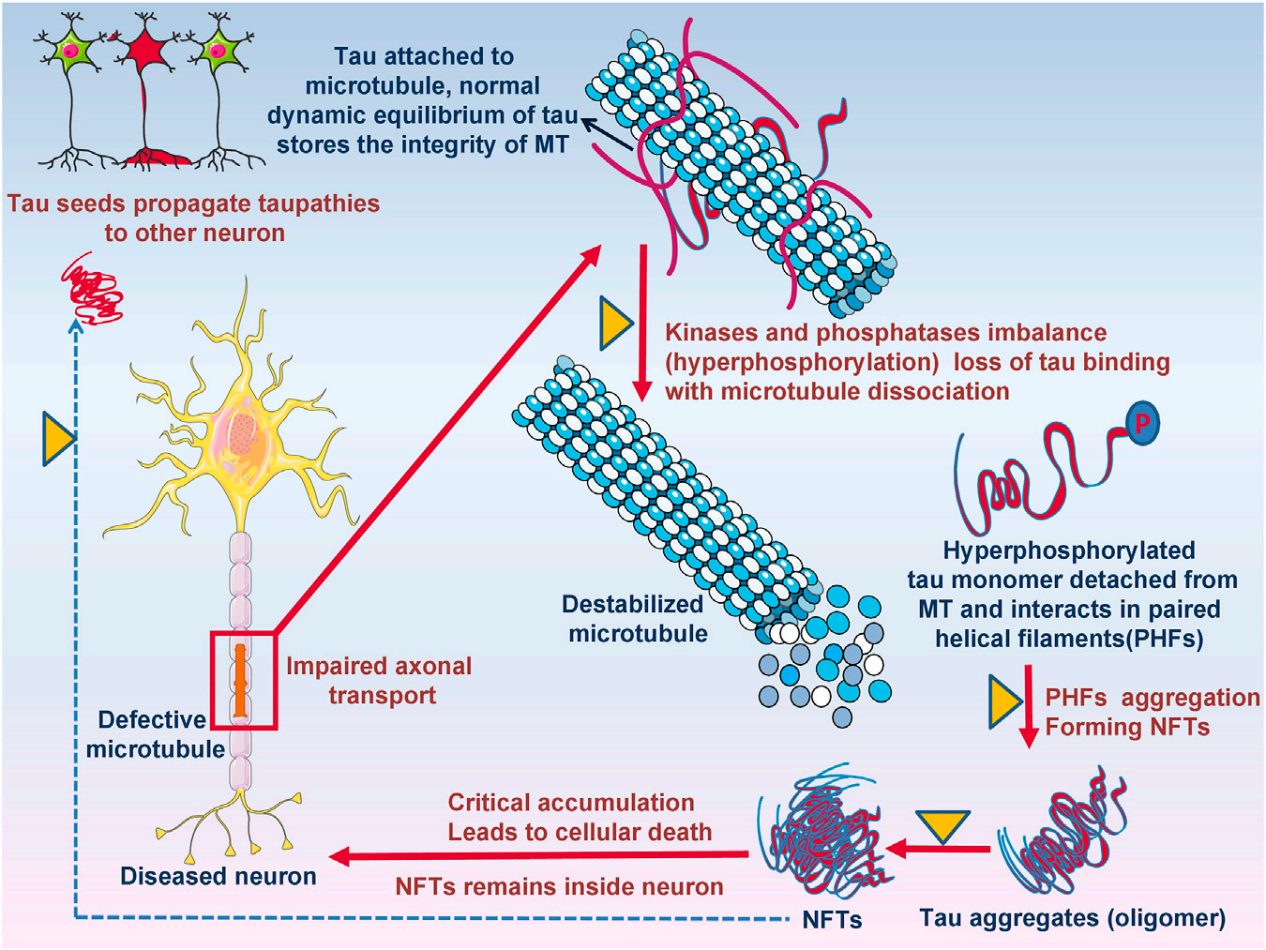
The potential role of ITCs in disease modification, targeting tau protein and its aggregation. Defective microtubules resulting in impaired axonal transport due to kinases and phosphatase imbalance resulting destabilized microtubule formation. Detached hyperphosphorylated tau monomers oligomerized and form NFTs leads to cellular death.

The brain of people suffering from Alzheimer’s disease shows remarkable accumulations of two neurotoxic proteins Aβ and tau (Cao et al., 2020). So far, several Alzheimer’s plaque and tau inhibitors from different sources are available they can target different mechanistic steps of fibril formation. One of the inhibitors that are widely used to stop protein aggregation is GLCs derivatives ITCs as they are consumed as a part of our daily diet (Lopez-Rodriguez et al., 2020). In Table 3, we have discussed some of the GLCs derived ITCs, proposed as the potential inhibitor of misfolded Aβ and tau aggregation and their induced toxicity by different mechanisms and modulation of multiple pathways (Figures 4, 5) as described earlier (Grande et al., 2020). Recent investigations suggested that they may directly interact with misfolded proteins during very early stages of the aggregation cascade by binding and stabilizing unfolded proteins and redirecting the aggregation pathways to form amorphous nontoxic fibrils, blocking seeding and further conformational changes that result in neurotoxicity and cell death.

**TABLE 3:** Beneficial effects of ITCs against pathological hallmarks and their neurotoxicity.

Plant/Compound	Mechanism of action	Pharmacological effectiveness	Test scale	References
*Against amyloid beta oligomerization and toxicity*				
*Wasabia japonica* (6-methylsulfinyl hexyl ITC)	Increased glutathione levels and ROS in hippocampus by Aβ_1-42_ injection were reduced	Neuroprotection against Aβ_1-42_ and ameliorates Aβ_1-42_ induced memory impairments	*in-vivo,* murine model, induced by intra cerebrovascular injection of Aβ_1-42_	Morroni et al. (2018)
Indole-3 carbinol (I3C)	High affinity molecular recognition and reduced Aβ fragments by heteromeric interaction	Reduced amyloid production	*in-vitro,* biochemical method	Cohen et al. (2006)
*Moringa oleifera* extract	Downregulated BACE1	Decreased Aβ production, rescued cognitive impairment and enhanced the reduced synaptic proteins synapsin, synapsophysin, PSD93 and PSD95	*in-vivo,* hyperhomocysteinemia (HHcY) induced AD model	Mahaman et al. (2018)
	Deactivated calpain by ↓ intracellular Ca 2+, reduced ca2+ signaling and prevent cell death	Decreased cytosolic cysteine protease caplain activity	*in-vivo,* hyperhomocysteinemia (HHcy) induced rat model (AD like pathology)	Mahaman et al. (2018)
	Increased Aβ immunoexpression was significantly abolished, sustained the brain-Zn content	Decreased the aggregation and accumulation of Aβ	*in-vivo,* ACR induced forty male Sprague Dawley rat treated with MO-ZnONP	Dahran et al. (2023)
Sulforaphane	Increased levels of HSP-70 co-chaperons and CHIP (Aβ metabolism influencers)	Reduced monomeric and polymeric forms of Aβ, but do not affect m-RNA expression, ameliorated memory deficits	*in-vivo*, triple transgenic mouse model (3×Tg-AD)	Li et al. (2018)
	Decreased oxidative stress and neuroinflammation (generator of Aβ)	Significantly inhibited Aβ aggregation, ameliorated neurobehavioral deficits peroxidation in brain	*in-vivo*, 6-month-old PS1V97L transgenic (Tg) mice	Zhang et al. (2015)
	Modulated the amyloid expression related markers, inhibited the overexpression of CDK5 in primary neurons	Reduced the Aβ_1-42_ deposition and related neurotoxicity	*in-vivo,* TgCRND8 transgenic mice model	Yang et al. (2023)
	Inhibited cathepsin-B and caspase-1 dependent NLRP3 inflammasome activation induced by Aβ monomers (1–42)	Reduced Aβ induced neurotoxicity	*in-vitro,* human THP-1 macrophages like cells	An et al. (2016)
	Alleviated several downstream pathological changes including oxidative stress and neuroinflammation	Significantly inhibited the generation of Aβ aggregates promotes spatial learning and memory	*in-vivo,* PS1V97L transgenic mice model	Hou et al. (2018)
*Against tau hyperphosphorylation and toxicity*				
*Moringa oleifera* extract	Not known	Decreased hyperphosphorylated tau at different sites (S-199, S-404, S-396 and T-231)	*in-vivo,* hyperhomocysteinemia (HHcy) induced rat model (AD like pathology)	Mahaman et al. (2018)
	Reduced sensory dysfunction and motor deficits, abolished immunoexpression of phosphorylated tau proteins	Reduced ACR induced neurotoxicity and tau proteins	*in-vivo,* ACR induced forty male Sprague Dawley rat treated with MO-ZnONP	Dahran et al. (2023)
Sulforaphane	Increased levels of HSP-70 co-chaperons and CHIP (Aβ metabolism influencers)	Reduced protein levels of tau and hyperphosphorylated tau, ameliorated memory deficits	*in-vivo,* triple transgenic mouse model (3×Tg-AD)	Lee et al. (2014)
	Suppressed phosphorylation of tau at specific sites, markedly suppressed the CDK5/p25	Reduced tau protein hyperphosphorylation in the brain and improved synaptic plasticity	*in-vivo,* TgCRND8 transgenic mice model	Yang et al. (2023)
	Altered phosphorylated tau at threonine 181 and serine991/202 distribution within astrocytes	Reduced hyperphosphorylated tau proteins in astrocytes under hypoglycaemic condition	*in vitro,* embryonic hippocampal rat astrocytes	Komiskey et al. (2022)
	Significantly inhibited hyperphosphorylated tau proteins at Ser396, Ser404 and Thr 205 site, enhanced the ration of p-GSK-3β(Ser9)/GSK-3β and p-Akt (Ser473)/Akt in hippocampus	Reduced the accumulation of phosphorylated tau in hippocampus and related toxicity	*in-vivo*,streptozotocin induced rat model	Yang et al. (2020)
	Significantly expressed the NDP52 induced by Nrf2 and facilitated clearance of p-tau proteins	Reduced the phosphorylated tau proteins	*in-vivo,* C57BL/6J mice model	Jo et al. (2014)

6-(Methylsulfinyl) hexyl isothiocyanate (6-MSITC) from *Wasabia japonica* was evaluated against amyloidosis in a murine mice model in which 6-MSITC was induced by intra cerebroventricular injection of Aβ_1-42_ oligomers. Behavioral analysis revealed that it reduced Aβ1-42 induced memory impairment in hippocampus tissues, increased ROS, and decreased glutathione levels following Aβ_1-42_ injection (Morroni et al., 2018). In another study, the authors observed that Aβ_25-35_ induced mitochondrial dependent cell death was blocked by SFN through Nrf2-associated manner (Brasil et al., 2023). Clinically, it inhibited Aβ, reduced its burden, and increased the expression of p75NTR in an intransgenic mouse model (Zhang et al., 2015). In another investigation, SFN was found to suppress Aβ deposition, improve cognition, and locomotor function in aluminum and D-galactose-induced mouse model (Zhang et al., 2017). It modulated the Aβ expression related markers followed CDK5 overexpression inhibition in primary neurons, further it reduced Aβ_1-42_ induced neurotoxicity and its deposition in TgCRND8-transgenic mice brains. It also suppressed tau phosphorylation at specific sites (Yang et al., 2023). It reduced and altered hyperphosphorylated tau proteins in embryonic hippocampal rat astrocytes under hypoglycaemic condition at Th 181 and Sr 991/202 within astrocytes (Komiskey et al., 2022). It induced NDP52 by Nrf2 and cleared the phosphorylated tauproteins in mice model (Jo et al., 2014).Through high affinity molecular recognition by heteromeric interaction of Aβ plaques, I3C were found to strongly reduce Aβ fibril formation as observed in microscopic examination by TEM analysis (Cohen et al., 2006).

*M.oleifera* is profoundly used against chronic diseases including AD. Mitochondrial apoptotic genes profile through GMC-ITC pre-treated SH-SY5Y neuronal cells revealed that it protect the cells against oxidative stress via apoptotic pathway, it significantly downregulate the expression of Bax, CASP3, CASP8, CASP9, Apaf-1, cyt-c, p-53 genes and upregulate Bcl2 gene in mitochondrial apoptotic signalling pathway (Jaafaru et al., 2019a). In another study GMC-ITC from the seeds of *M. oleifera* significantly decreased the expression of BACE1, APP and increased the expression of MAPT tau genes in H_2_O_2_ induced cytotoxic neuroblastoma cell (SH-SY5Y) (Jaafaru et al., 2019b). It decreases Aβ production and enhance the synaptic proteins in HHcY induced AD model bydown regulating BACE1. It also played crucial role in Ca^2+^ homeostasis, as it deactivated calpain by decreasing intracellular Ca^2+^ resulting cytosolic protease calpain activity reduction in HHcY induced rat model (Mahaman et al., 2018). In another study conducted on MO-ZnONP treated Sprague Dawley rat model it reduced the Aβ accumulation and helped in sustained brain-Zn content (Dahran et al., 2023).

## 5 Conclusion

GLCs derived ICTs are important bioactive natural products that are found in many Brassicaceae plants and few plants from other families. *In vitro* and animal studies have reported their beneficial effects in neuroprotection and they are reported to enhance cellular metabolism, nourish brain cells, and reduce risk factors associated with neurodegeneration. ITCs inhibit inflammatory mediators, oxidative stress, cellular stress signaling, and improve behavioural measures. They also easily cross the blood brain barrier to interact with particular targets implicated in AD pathogenesis. However, there is no sufficient clinical evidence to prove these effects in humans. Future studies should focus to evaluate their pharmacokinetic parameters and effectiveness in humans.

## Abbreviations

AD, Alzheimer’s disease; AITC, allylisothiocyanate; APP, amyloid precursor protein; Aβ, amyloid beta; BBB, blood brain barrier; ER, erucin; GLCs, glucosinolates; I3C, indole 3 carbinol; IL-1β, interleukin-1β; iNOS, inducible nitric oxide synthase; ITCs, isothiocyanates; MAPK, mitogen activated protein kinase; MO, *Moringa oleifera;* NF-kβ, nuclear factor kappa B; NFT, neurofibrillary tangles; PEITC, phenethylisothiocyanate; p-tau, phosphorylated tau; ROS, reactive oxygen species; SFN, sulforaphan; TNF-α, tumor necrosis factor- α.
